# Transcriptome profiling and RNA-Seq SNP analysis of reniform nematode (*Rotylenchulus reniformis*) resistant cotton (*Gossypium hirsutum*) identifies activated defense pathways and candidate resistance genes

**DOI:** 10.3389/fpls.2025.1532943

**Published:** 2025-02-19

**Authors:** Martin J. Wubben, Sameer Khanal, Amanda G. Gaudin, Franklin E. Callahan, Jack C. McCarty, Johnie N. Jenkins, Robert L. Nichols, Peng W. Chee

**Affiliations:** ^1^ Genetics and Sustainable Agriculture Research Unit, USDA-ARS, Crop Science Research Laboratory, Mississippi State, MS, United States; ^2^ Department of Crop and Soil Sciences, University of Georgia, Tifton, GA, United States; ^3^ Cotton Incorporated, Cary, NC, United States

**Keywords:** cotton, reniform nematode, resistance genes, transcriptome, RNA-seq

## Abstract

The reniform nematode (*Rotylenchulus reniformis* Linford & Oliveira) is a serious pathogen of Upland cotton (*Gossypium hirsutum* L.) wherever it is grown throughout the United States. Upland cotton resistance to *R*. *reniformis* derived from the *G*. *barbadense* accession GB713 is largely controlled by the *Ren^barb2^* locus on chromosome 21. *Ren^barb2^* has proven useful as a tool to mitigate annual cotton yield losses due to *R*. *reniformis* infection; however, very little is known about the molecular aspects of *Ren^barb2^*-mediated resistance and the gene expression changes that occur in resistant plants during the course of *R*. *reniformis* infection. In this study, two nearly isogenic lines (NILs), with and without the *Ren^barb2^* locus, were inoculated with *R*. *reniformis* and RNAs extracted and sequenced from infected and control roots at 5-, 9-, and 13-dai (days after inoculation). A total of 966 differentially expressed genes (DEGs) were identified in the resistant NIL while 133 DEGs were discovered from the susceptible NIL. In resistant plants, biological processes related to oxidation-reduction reactions and redox homeostasis were enriched at each timepoint with such genes being up-regulated at 5- and 9-dai but then being down-regulated at 13-dai. DEGs associated with cell wall reinforcement and defense responses were also up-regulated at early timepoints in resistant roots. In contrast, in susceptible roots, defense-related gene induction was only present at 5-dai and was comprised of far fewer genes than in the resistant line. ERF, WRKY, and NAC transcription factor DEGs were greatly enriched at 13-dai in resistant roots but were absent in the susceptible. Cluster analysis of resistant and susceptible DEGs revealed an ‘early’ and ‘late’ response in resistant roots that was not present in the susceptible NIL. SNP analysis of transcripts within the *Ren^barb2^* QTL interval identified five genes having non-synonymous mutations shared by other *Ren^barb2^* germplasm lines. The basal expression of a single candidate gene, Gohir.D11G302300, a CC-NBS-LRR homolog, was ~3.5-fold greater in resistant roots versus susceptible. These data help us to understand the *Ren^barb2^*-mediated resistance response and provides a short list of candidate resistance genes that potentially mediate that resistance.

## 1 Introduction

Cotton (*Gossypium* spp.) is the primary source of natural textile fiber in the world and contributes approximately $21 billion to the U.S. economy annually through products and services. More than 97% of U.S. cotton grown is of the upland type (*G*. *hirsutum*). Upland cotton is an excellent host for many plant-parasitic nematode (PPN) species where infection by these root pathogens leads to significant annual yield losses. One of the most important PPNs of cotton is the reniform nematode (*Rotylenchulus reniformis* Linford & Oliveira), a sedentary semi-endoparasitic species that is distributed throughout the southeastern United States and Texas where it causes stunting, wilting, delayed maturity, and root rot of upland cotton (Singh et al., 2023). Annual cotton yield losses to *R*. *reniformis* have averaged around 2% over the past decade; however, losses have been as high as 8% in Mississippi and Alabama and nearly 50% in individual fields (Singh et al., 2023).

Host root infection by *R*. *reniformis* is accomplished by the vermiform adult female nematode at any point within the root system. Penetration of the root epidermis and movement through the cortex is done by mechanical force of the nematode’s stylet, a protrusible, hollow mouth-spear, coupled with the secretion of cell wall degrading enzymes originating in the esophageal glands and passing through the stylet opening (Mitchum et al., 2013). As an obligate biotrophic parasite, *R*. *reniformis* establishes a feeding site comprised of multiple fused cells within the host root tissue called a syncytium. The syncytium begins as an initial nurse cell, usually endodermal, that eventually incorporates surrounding pericyclic cells via partial cell wall dissolution to form the syncytium (Robinson, 2007). Syncytium formation and maintenance is accomplished via the action of effector proteins secreted by the nematode through the stylet that alter host cell gene expression and metabolism and also work to suppress the host defense response (Mitchum et al., 2013). Sedentary females are fertilized by non-feeding male nematodes and lay 60-200 eggs within a gelatinous matrix. Second-stage juveniles hatch from the eggs, undergo three successive molts in the soil, and emerge as either infective vermiform females or male nematodes, thereby completing the lifecycle. Under optimal conditions lifecycle completion from egg-to-egg can take as little as 17 days, thus allowing for many generations to occur during a growing season (Robinson, 2007). Until recently, control of *R*. *reniformis* by cotton producers was limited to nematicide treatment coupled with or without crop rotation of a non-host to reduce field populations (Robinson, 2007). Currently, a handful of commercial varieties have been released that carry resistance to *R*. *reniformis* and these have been used with good success to control the nematode in the field (Turner et al., 2023).

For many of the *R*. *reniformis* resistant cultivars being used by producers, a main component of the resistance is derived from the wild *G*. *barbadense* accession GB713. GB713 was discovered as being resistant to *R*. *reniformis*, reducing nematode reproduction by approximately 95% compared to susceptible plants (Robinson et al., 2004). QTL mapping using F_2_ and back-cross populations identified three resistance loci: *Ren^barb1^* and *Ren^barb2^* on chromosome 21 and *Ren^barb3^* on chromosome 18 (Gutierrez et al., 2011). Using nearly isogenic lines (NILs), it was later shown that *Ren^barb1^* was a false QTL and that *Ren^barb2^* alone conferred ~ 80% of the GB713-derived resistance phenotype (Wubben et al., 2017; Gaudin et al., 2020). *Ren^barb2^* NILs showed a substantial decrease in the number of *R*. *reniformis* sedentary females that were able to form egg masses, indicating that resistance acted early in the infection process and most likely worked to disrupt feeding site formation (Wubben et al., 2017). A similar conclusion was drawn by Stetina (2015) who tracked *R*. *reniformis* infection and development in GB713 directly. That study showed that at as early as 5 days after inoculation, a decreased number of nematodes was apparent on GB713 roots versus the susceptible control and that females that became sedentary on GB713 lagged in their development compared to females on susceptible roots (Stetina, 2015).

Investigations into the molecular nature of cotton resistance to *R*. *reniformis* are limited. A transcriptome analysis of two cotton germplasm lines, one hypersensitive to *R*. *reniformis* (LONREN-1) and another expressing GB713-derived resistance (BARBREN-713), identified a number of induced defense signaling pathways such as cell wall biogenesis, redox reactions, and secondary metabolism (Li et al., 2015). This study also identified multiple LRR-like and NBS-LRR domain-containing genes that were differentially expressed in resistant plants that were physically located near known QTL intervals (Li et al., 2015). While this study collected infected roots at multiple times post inoculation, the samples were pooled for RNA extraction; consequently, the temporal assessment of gene expression changes in resistant plants was not analyzed (Li et al., 2015). A second recent study examined the resistance responses of a *R*. *reniformis* resistant *G*. *arboreum* germplasm line along with the GB713 accession and the *G*. *barbadense* accession TX110 at two timepoints after inoculation (Feng et al., 2024). This study identified genes involved in plant defense and systemic acquired resistance that were induced at the earlier timepoint in GB713 (Feng et al., 2024). The induction of systemic acquired resistance in LONREN-1 plants following *R*. *reniformis* infection has also been demonstrated (Aryal et al., 2011).

In the present study, we utilized two upland cotton NILs that differed only in the presence or absence of the GB713-derived *Ren^barb2^* QTL to address two primary objectives: (i) identify the signaling and metabolic pathways in cotton roots that participate in *R*. *reniformis* resistance as mediated by the *Ren^barb2^* QTL and (ii) use RNA-Seq data to identify *G*. *barbadense*-specific SNPs in genes within the *Ren^barb2^* QTL interval, thereby, generating a set of candidate resistance genes for marker development and functional studies.

## 2 Materials and methods

### 2.1 Nematode culture and plant materials

A reniform nematode (*Rotylenchulus reniformis*) culture was maintained on the susceptible cotton line M8 in a growth chamber. Eggs used for inoculum in experiments were collected from infected M8 roots by sodium hypochlorite washing according to the method of Hussey and Barker (1973). Nearly-isogenic lines (NILs) fully susceptible to *R*. *reniformis* or having the GB713-derived *Ren^barb2^* resistance QTL had previously been developed and characterized by our research group (Wubben et al., 2017).

Seeds of resistant and susceptible NILs were scarified, imbibed in water at 30°C for four hours, and then germinated in paper towels overnight at 30°C. Germinated seeds having ~ 0.5 cm radicles were sown into 20 cm Cone-tainers containing a 1:7 mix of autoclaved silty loam soil:sand. Inoculated Cone-tainers received ~ 5,000 *R*. *reniformis* eggs one-day after planting whereas nothing was added to control plants. Cone-tainers were arranged in rows of three within racks such that one row equaled a single biological replicate. Upon root tissue harvest, the root systems of three plants within each row were pooled for RNA extraction. Three biological replicates of each NIL (resistant, susceptible) × timepoint (5-, 9-, 13-days after inoculation) × treatment (control, inoculated) were arranged in racks in a completely randomized design. Plants were grown in a Percival PGC-9/2 growth chamber at 30°C on a 16 hr. day/8 hr. night schedule. Extra root samples were taken at each timepoint and stained with acid fuchsin to visualize the extent of *R*. *reniformis* infection and stages of development. Root staining with acid fuchsin was accomplished according to Wubben et al. (2020). Pictures of infected roots were taken with a digital camera mounted on a Nikon SMZ1500 stereomicroscope.

### 2.2 RNA extraction and sequencing

Root tissues were washed free of soil, blotted dry, wrapped in aluminum pouches, flash-frozen in liquid nitrogen, and stored at -80°C until RNA extraction. Total RNA was isolated by grinding the root samples under liquid nitrogen with a mortar and pestle and extracting the RNA via the hot borate method as previously described (Wubben et al., 2019). Purity and concentration of total RNA was determined using a NanoDrop 1000 spectrophotometer. A total of (24) RNA samples representing two of the three biological replicates each of NIL × timepoint × treatment was submitted to the Georgia Genomic Facility (University of Georgia). Sequencing libraries were made using the Kapa Stranded RNA-Seq Kit (Roche Inc., Indianapolis, IN) and 150-bp paired-end reads were generated using a NextSeq PE150 High Output flow cell platform (Illumina Inc., San Diego, CA). Sequence quality was assessed using FastQC (Andrews, 2010). Low-quality bases and adapter sequences were trimmed from paired reads using Trimmomatic v0.30 (Bolger et al., 2014).

### 2.3 Transcriptome assembly and DEG analysis

The *Gossypium hirsutum* (AD1) Genome-Texas Interim release UTX-JGI v1.1 (available via www.cottongen.org) was used for sequence read alignment. Cotton transcriptome assembly was accomplished using the Galaxy web platform via public server at usegalaxy.org (Afgan et al., 2016) using the following built-in functions: sequence alignment was done using HISAT2 v2.1.0 (Kim et al., 2015) and transcriptome assembly using Stringtie v1.3.3 (Pertea et al., 2015). A total of 201,260,466 (~200 million) paired-end reads were generated, of which 159,566,283 (~160 million; ~79%) were concordantly mapped (using HISAT2) to the reference genome (**Supplementary Figure S1**, **Supplementary Table S1**). Based on overall alignment (concordant, discordant, or one of a mate-pair match), approximately 91% of sequences aligned to the reference genome. All catalogued exons (396,608) and loci (66,522) in the reference assembly/annotation were represented in the unified transcriptome (high sensitivity) reconstructed from 24 transcriptome assemblies (using StringTie’s merge), while 179,260 (27.2%) exons corresponding to 89,797 (56.9%) loci were specific to this dataset.

Transcriptome analysis was done using GffCompare (https://github.com/gpertea/gffcompare), gene count using htSeq-count (Anders et al., 2015), and differential expression (DEG) using DESeq2 (Love et al., 2014). For DEG analysis, an adjusted P-value (FDR) of ≤ 0.05 was calculated using the approach of Benjamini and Hochberg (1995). A significant fold-change (FC) in transcript expression was determined at 2-FC (log_2_FC > 1.0 or < -1.0). DEG contrasts were made between infected and control at each timepoint for resistant and susceptible cotton NILs. Gene ontology (GO) and KEGG pathway enrichment of genes was accomplished using the data fetch and enrichment tool at CottonFGD (cottonfgd.org) (Zhu et al., 2017). Hierarchical cluster analysis of DEGs was done using the ‘Expression’ tool at www.heatmapper.ca/expression with the settings average linkage for clustering method and Euclidean distance measurement method (Babicki et al., 2016).

### 2.4 Quantitative RT-PCR

Total RNA used for transcriptome sequencing was also used for qRT-PCR. Approximately 1 µg of total RNA was used for genomic DNA digestion and cDNA synthesis using the iScript^Tm^ gDNA Clear cDNA Synthesis Kit (Bio-Rad, Carlsbad, CA). cDNA reactions were diluted 1:10 to use as template in qRT-PCR reactions. Reactions were performed on a Bio-RAD CFX^TM^ Opus 96 Real-Time PCR Detection System. Reaction volumes were 10 µl and comprised as follows: 5.0 µl 2X iQTM SYBR^®^ Green Supermix (Bio-Rad), 0.25 µM primer, 1 µl cDNA template, and deionized water. Three technical reps of each primer × template combination were performed. GhUBQ14 Ct values were used for expression normalization. Two-sample t tests (P ≤ 0.05) were performed on the average normalized ΔCt value of three biological replicates of inoculated versus control samples. Mean fold-change in expression was converted to 2^-ΔΔCt^ for data presentation. Primers used in qRT-PCR experiments are provided in **Supplementary Table S2**.

## 3 Results

### 3.1 *Rotylenchulus reniformis* infection and development on resistant and susceptible NILs

**Figure 1:**
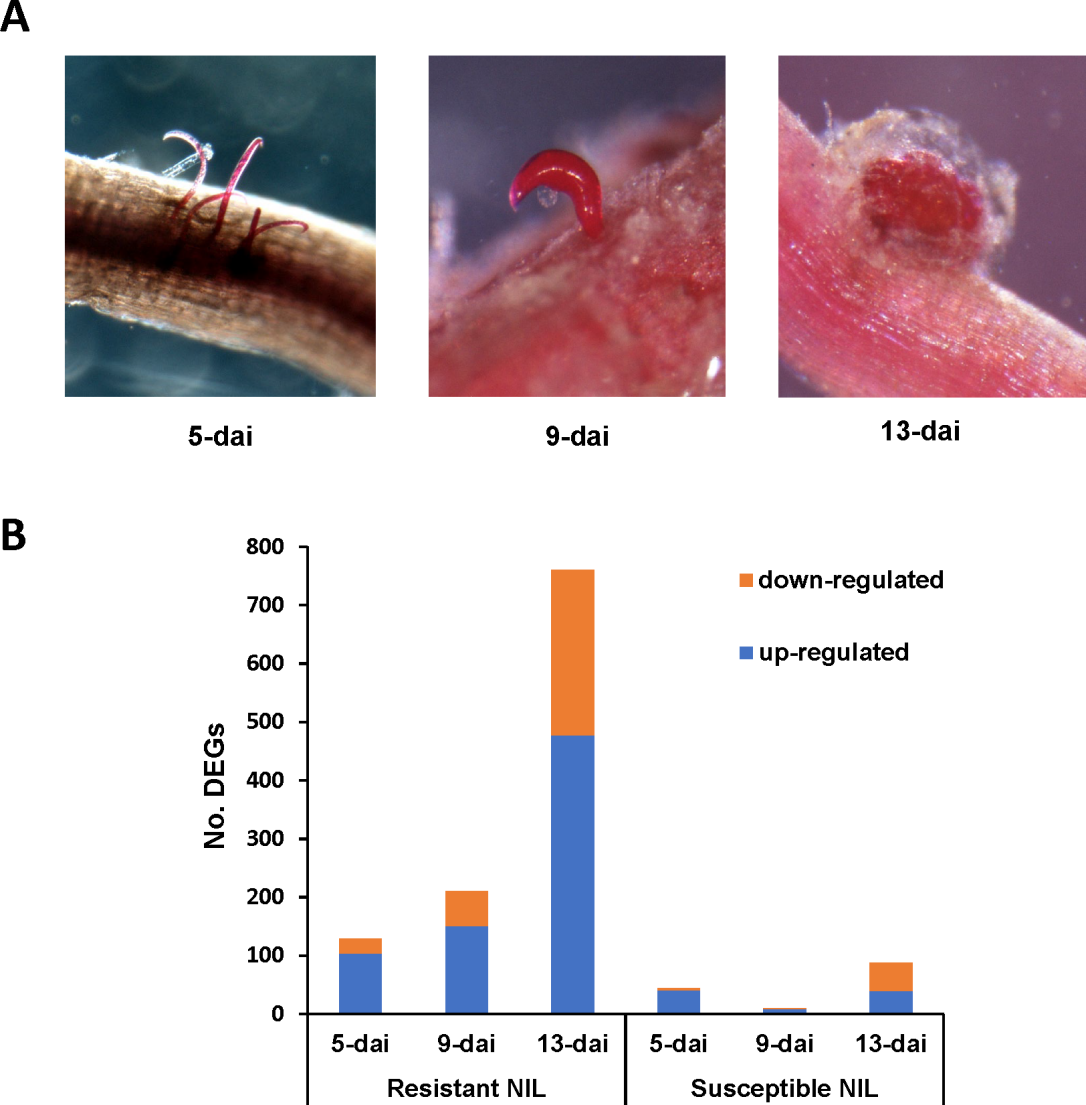
**(A)** Progression of *Rotylenchulus reniformis* development on the susceptible cotton near-isogenic line (NIL) at 5-, 9-, and 13-dai (days after inoculation). Sedentary females are stained red by acid fuchsin. **(B)** Numbers of up- and down-regulated differentially expressed genes (DEGs) in *R*. *reniformis* resistant and susceptible NILs at 5-, 9-, and 13-dai.

Resistant and susceptible NILs showed clear differences in *R*. *reniformis* infection levels and rate of female development over the course of the experiment and in particular at the 9-dai and 13-dai timepoints. At 5-dai, both resistant and susceptible NILs showed roughly equivalent levels of infection as evidenced by the presence of vermiform sedentary female *R*. *reniformis* protruding from the root epidermis (**Figure 1A**). By 9-dai, sedentary females had attained their characteristic kidney-shape; however, the susceptible line showed a much greater number of females compared to resistant roots, and in many instances the females on susceptible roots had begun to produce egg masses, whereas female development on resistant roots lagged behind (**Figure 1A**). By 13-dai, sedentary females on resistant and susceptible root systems had formed egg masses; however, far fewer females were observed on resistant roots versus susceptible (**Figure 1A**). These observations indicate an early resistance reaction occurring shortly after infection in the resistant NIL roots that appears to inhibit feeding site establishment and female development.

### 3.2 Identification of differentially expressed genes

To obtain a global view of gene expression changes in resistant and susceptible plants during *R*. *reniformis* infection, RNA-Seq was performed on control and infected roots at 5-, 9-, and 13-dai. A total of 1,099 unique DEGs were identified with the vast majority of DEGs coming from resistant plant roots (**Figure 1B**). In resistant roots, up- and down-regulated DEGs increased over time with total DEGs being 129, 210, and 760 at 5-, 9-, and 13-dai, respectively, with up-regulated DEGs always being in the majority. In contrast, in susceptible roots, only 44, 10, and 87 DEGs were detected at the same timepoints with up- and down-regulated DEGs being split near equally at 13-dai (**Figure 1B**). These findings demonstrate a significantly stronger transcriptional response, both positively and negatively, occurred in resistant roots versus susceptible following *R*. *reniformis* infection.

**Figure 2:**
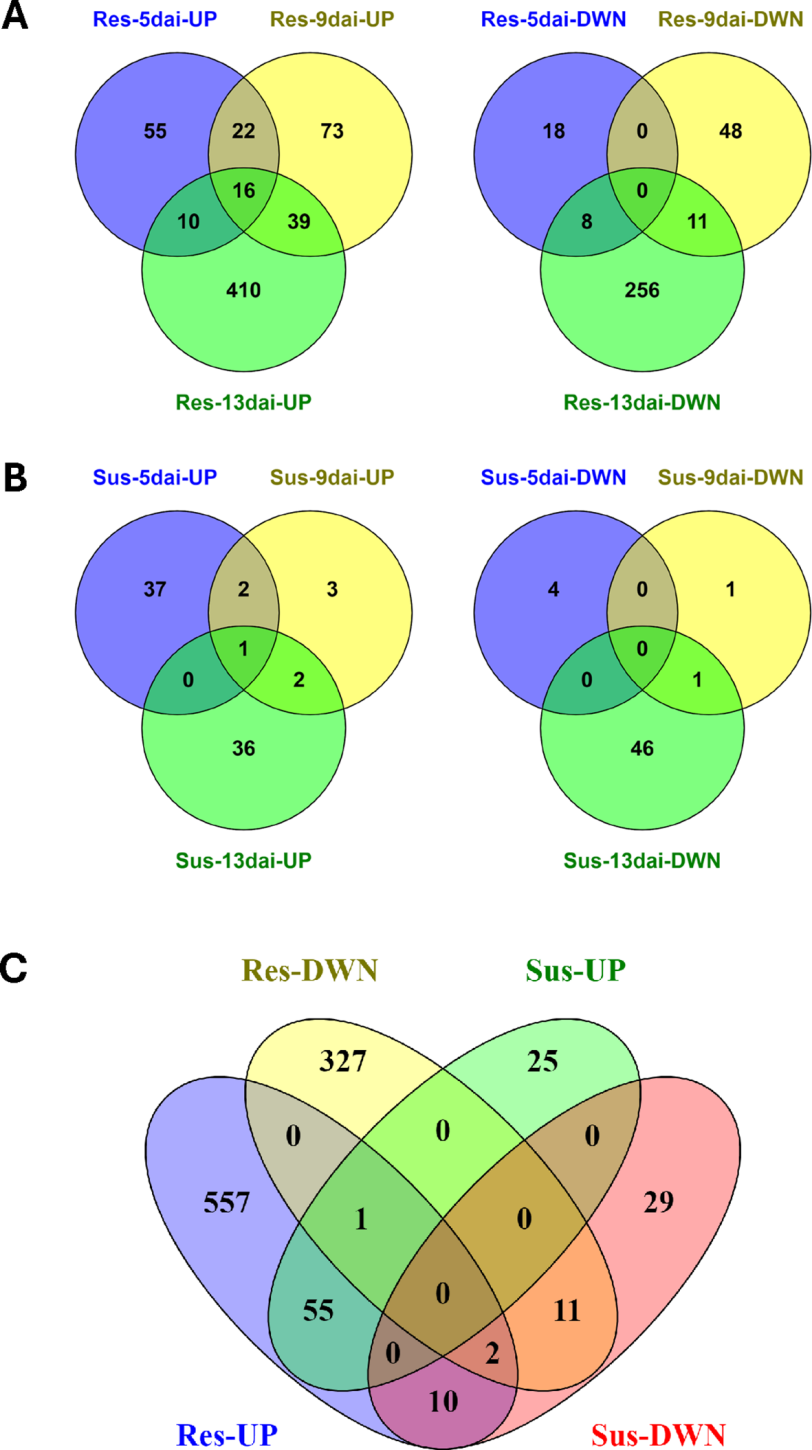
Venn diagrams showing shared up- and down-regulated DEGs at 5-, 9-, and 13-dai (days after inoculation) in *Rotylenchulus reniformis* resistant **(A)** and susceptible **(B)** cotton near-isogenic lines (NILs). Venn diagram showing shared up- and down-regulated DEGs across all timepoints following *R*. *reniformis* inoculation in resistant and susceptible NILs **(C)**.

The majority of DEGs in resistant and susceptible roots were restricted to single timepoints. Among up-regulated DEGs in resistant roots, 87/625 appeared in more than one timepoint and only 16 genes were up-regulated across all timepoints (**Figure 2A**). Similarly, among down-regulated DEGs in resistant roots, 19/341 appeared in more than one timepoint; however, no DEGs were down-regulated across all timepoints (**Figure 2A**). In susceptible roots, 5/81 up-regulated DEGs appeared in more than one timepoint and only a single gene was up-regulated across all timepoints (**Figure 2B**). This gene also happened to be up-regulated across all timepoints in resistant roots, i.e., Gohir.D10G155200, and encodes a putative early nodulin-75-like protein (**Table 1**). A total of 52 DEGs were down-regulated in susceptible roots with only a single gene appearing at more than one timepoint (**Figure 2B**). A comparison between up- and down-regulated DEGs between resistant and susceptible roots identified some commonalities in gene expression. For example, approximately 63% of susceptible up-regulated DEGs were also found to be up-regulated in resistant roots for at least a single timepoint (**Figure 2C**). Likewise, approximately 22% of susceptible down-regulated DEGs were also down-regulated in resistant roots (**Figure 2C**). In contrast, a group of 10 genes was identified that showed up-regulation in resistant roots but down-regulation in the susceptible line (**Figure 2C**). Among these 10 genes were multiple defense-related proteins, including laccases, nematode-resistance protein-like, ERF5-like protein, phosphatase 2C, and phospholipase. Sixteen (16) genes were induced in resistant roots across all three timepoints (**Table 1**). In many instances, genes were also induced in the susceptible genotype at one or more timepoints but not all three with the exception of two early-nodulin-75-like homologs that were induced at all timepoints in both lines. Many of the genes were defense-related such as chitinase, WRKY transcription factor, lipoxygenase, and MIC3. MIC3 was induced in resistant and susceptible plants at 5-DAI, but induction only continued in resistant plants through 9- and 13-dai. A similar pattern of expression between resistant and susceptible roots was observed for chitinase, WRKY5, lipoxygenase, and two uncharacterized proteins (**Table 1**). These findings suggest that an initial response to *R*. *reniformis* infection occurred in the roots of the susceptible NIL but there was no sustained resistance response as in the resistant root tissues.

**Table 1:** Cotton genes induced at all timepoints in *Ren^barb2^* resistant plants inoculated with reniform nematode.

Gene ID	5-dai^†^	9-dai^†^	13-dai^†^	TrEMBL Hit (E-value)
Gohir.A01G172800	1.46*	1.88	2.07	Class I chitinase OS=Gossypium hirsutum (0.0)
Gohir.A01G189900	1.78	1.23	2.68	Alcohol dehydrogenase A OS=Gossypium raimondii (0.0)
Gohir.A02G003000	1.71*	1.87	1.63	WRKY transcription factor 5 OS=Gossypium hirsutum (0.0)
Gohir.A03G158000	1.25	1.35	1.14*	(R,S)-reticuline 7-O-methyltransferase-like OS=Gossypium hirsutum (0.0)
Gohir.A08G165000	1.26	1.52	1.38	histone-lysine N-methyltransferase, H3 lysine-9 specific SUVH6-like OS=Gossypium hirsutum (0.0)
Gohir.A08G210400	1.65*	1.24	1.52	Lipoxygenase OS=Gossypium hirsutum (0.0)
Gohir.A10G112100	2.04*	2.19*	1.88*	early nodulin-75-like OS=Gossypium hirsutum (7.11E-65)
Gohir.A13G167800	1.25	1.02	1.39	probable low-specificity L-threonine aldolase 1 OS=Gossypium hirsutum (0.0)
Gohir.D01G163800	1.28	1.41	1.97	Cytokinin riboside 5’-monophosphate phosphoribohydrolase OS=Gossypium arboreum(1.9E-154)
Gohir.D05G366400	1.83*	1.59	2.11	MIC-3 OS=Gossypium tomentosum (9.48E-97)
Gohir.D06G183800	1.94*	1.44	1.43	Uncharacterized protein OS=Gossypium raimondii (4E-154)
Gohir.D10G006400	2.22	1.26	1.80	Berberine bridge enzyme-like 7 OS=Arabidopsis thaliana (1.6E-163)
Gohir.D10G155200	2.57*	2.05*	2.40*	early nodulin-75-like OS=Gossypium hirsutum (4.8E-102)
Gohir.D11G295000	1.24*	2.53*	1.54	Uncharacterized protein OS=Gossypium raimondii (9.14E-24)
Gohir.D12G106100	1.14	1.50	1.54	Subtilisin-like protease SBT1.1 OS=Arabidopsis thaliana (0.0)
Gohir.D12G266500	1.24	1.91	1.16	Uncharacterized protein OS=Gossypium raimondii (1.4E-150)

*Induction to similar level present in susceptible plants inoculated with reniform nematode.

^†^Log_2_(fold-change) versus control roots from same genotype at same timepoint (dai – days after inoculation).

### 3.3 Hierarchical cluster analysis

**Figure 3:**
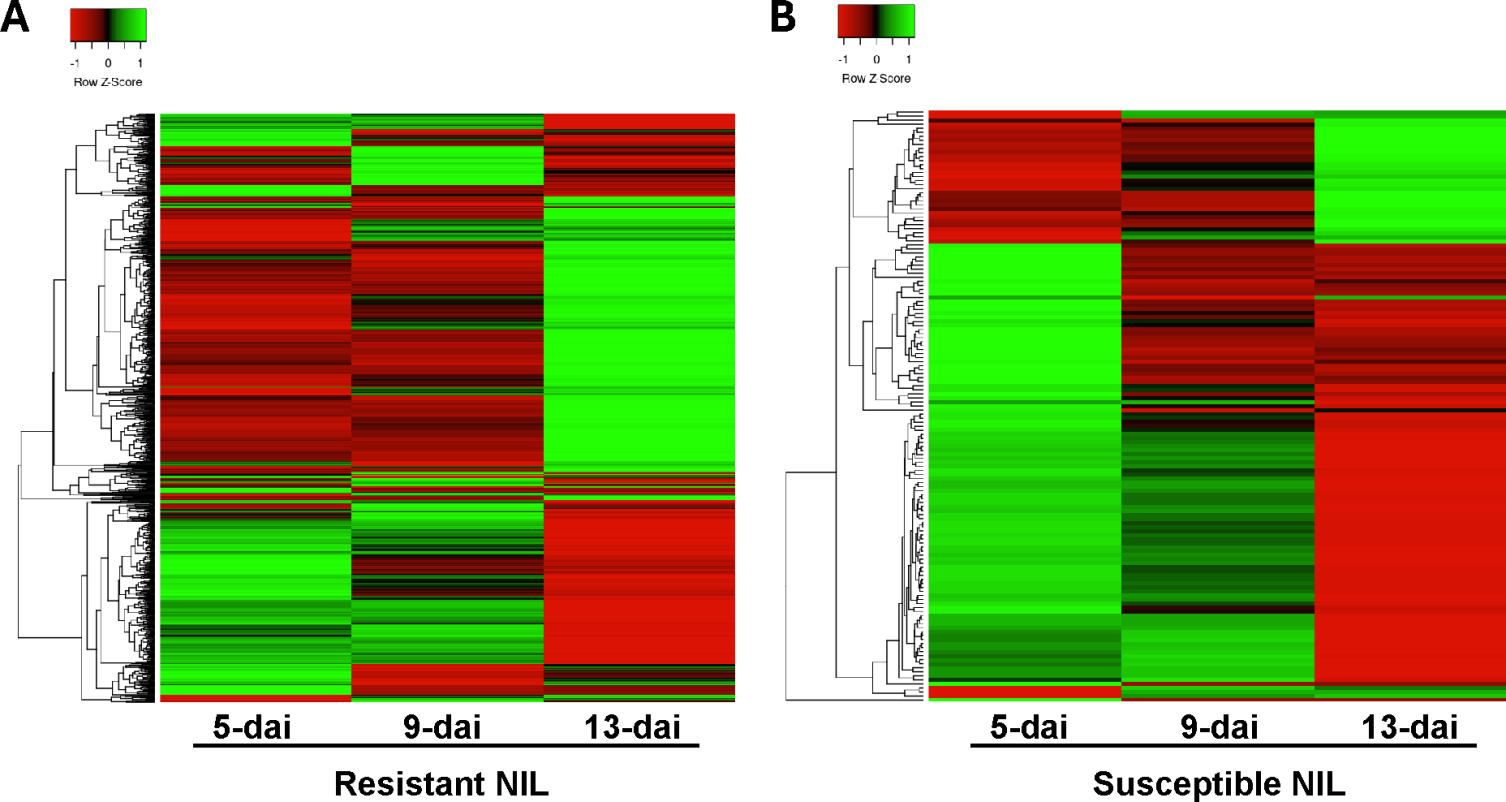
Hierarchical cluster analysis of differentially expressed genes in **(A)** resistant (n=966) and **(B)** susceptible (n=133) nearly-isogenic lines at 5-, 9-, and 13-dai (days after inoculation) with *Rotylenchulus reniformis*. Green shading indicates up-regulated gene expression and red-shading indicates down-regulated gene expression.

Hierarchical clustering of all resistant and susceptible DEGs across all timepoints was performed to provide a comprehensive perspective of DEG expression patterns (**Figure 3**). In resistant roots, two predominant DEG expression patterns were apparent: (i) genes being induced early in the *R*. *reniformis* infection process at 5- and 9-dai but then returning to baseline expression levels or being down-regulated by 13-dai and (ii) genes staying at baseline levels early in the infection process but then being dramatically up-regulated at 13-dai (**Figure 3A**). Interestingly, these two trends were observed in susceptible roots, only with far fewer genes compared to resistant plants (**Figure 3B**). These observations indicate a biphasic response in resistant roots, i.e., an ‘early’ and ‘late’ response, that happens to be much stronger and with more genes in resistant versus susceptible plants.

### 3.4 Gene ontology enrichment analyses

GO enrichment analysis was performed on DEGs from resistant and susceptible roots to identify biological processes that were overrepresented at each timepoint. Due to the low number of DEGs in susceptible roots, only a handful of GO annotations were identified. At 5-dai, oxidation-reduction process (GO:0055114) and carbohydrate metabolism (GO:0005975) were enriched in susceptible roots with 10 and 3 genes, respectively, that were up-regulated (**Table 2**). These genes would be part of the early response clade in **Figure 3B** with representatives including peroxidase, lipoxygenase, and other oxygenases with varying cellular functions.

**Table 2:** Gene counts of GO biological process annotations up-regulated (green shading) and/or down-regulated (red shading) in resistant and susceptible roots at 5-, 9-, and 13-days after inoculation (dai) with reniform nematode.

Accession	Biological Process	Resistant NIL			Susceptible NIL		
		5-dai	9-dai	13-dai	5-dai	9-dai	13-dai
GO:0055114	oxidation-reduction	24/0	29/7	0/36	10/0		0/5
GO:0046274	lignin catabolism	4/0					0/4
GO:0045454	cell redox	3/0					
GO:0005975	carbohydrate metabolism	6/0	10/5	14/0	3/0		
GO:0006508	proteolysis	4/0					
GO:0008272	sulfate transport	0/3					
GO:0019953	sexual reproduction		3/0				
GO:0006979	oxidative stress response		5/0	0/8			
GO:0006952	defense response		3/0	4/0			
GO:0006073	glucan metabolism		0/4				
GO:0042546	cell wall biogenesis		0/3				
GO:0010411	xyloglucan metabolism		0/3				
GO:0006810	transport		0/7	0/16			
GO:0055085	transmembrane transport	0/3	0/3	0/13			
GO:0006355	regulation of transcription			62/0			
GO:0016567	protein ubiquitination			14/0			
GO:0006470	protein dephosphorylation			12/0			
GO:0006487	protein N-linked glycosylation			3/0			
GO:0006950	response to stress			5/0			
GO:0006486	protein glycosylation			3/0			
GO:0006542	glutamine biosynthesis			0/6			
GO:0006807	nitrogen metabolism			0/6			
GO:0009664	cell wall organization			0/4			
GO:0000160	signal transduction			0/5			
GO:0030244	cellulose biosynthesis			0/3			

Substantially more GO annotations were enriched in resistant versus susceptible roots (**Table 2**). Biological processes related to oxidation-reduction and cellular redox homeostasis were heavily represented in resistant roots at 5-dai. DEGs with roles in oxidation-reduction continued to be up-regulated at 9-dai; however, down-regulation of this process also began to occur at this timepoint. By 13-dai, oxidation-reduction DEGs were wholly down-regulated in resistant roots. In contrast, DEGs associated with regulation of transcription (GO:0006355) and various protein secondary modifications, e.g., ubiquitination (GO:0016567), dephosphorylation (GO:0006470), and glycosylation (GO:0006487), were absent at early timepoints but strongly up-regulated at 13-dai. Genes involved in carbohydrate metabolism (GO:0005975) were up-regulated at each time-point with some down-regulation at 9-dai. Processes associated with cell wall fortification such as lignin catabolism (GO:0046274) were up-regulated early at 5-dai but then down-regulated by 9-dai (cell wall biogenesis; GO:0043546) and 13-dai (cell wall organization; GO:0009664). Enrichment by molecular function showed an overabundance of genes involved in DNA binding and transcription factor activity in resistant roots (**Supplementary Figure S2**). Genes with functions in oxidoreductase activity or other catalytic activity were also heavily represented in resistant roots (**Supplementary Figure S2**). Enrichment by cellular component further highlighted the resistance/defense nature of gene activity in resistant roots in that cellular spaces ‘outside’ of the cell or related to signaling or secretory processes were overrepresented such as the plasma membrane, apoplast, exocyst, and extracellular space (**Supplementary Figure S2**). In susceptible roots, only the apoplast was enriched, likely representing an early, yet unsustained, response to *R*. *reniformis* infection.

The number of KEGG-enriched pathways increased over time in resistant roots with metabolic pathways (ko1100) and biosynthesis of secondary metabolites (ko1110) predominating at each individual timepoint (**Supplementary Table S3**). Host plant resistance to nematodes many times involves the activity of compounds that have nematicidal or nemastatic activity and are the products of secondary metabolic pathways (Sato et al., 2019). Phenylpropanoid biosynthesis (ko00940) and plant hormone signal transduction (koko04075) became apparent at 9-dai and remained through 13-dai. At 13-dai the aforementioned pathways were joined by a host of others including ethylene biosynthesis (M00368), plant-pathogen interaction (ko04626), and nitrogen metabolism (ko00910), indicating a large change in transcriptional regulation of multiple signaling and biosynthetic pathways in resistant roots. In contrast, susceptible roots were enriched only at 13-dai largely categorized as metabolic pathways and secondary metabolite biosynthesis which likely reflects the nutrient-sink nature of the syncytium in susceptible plant roots and indicate a compatible interaction with the nematode.

### 3.5 Oxidation-reduction processes in *R*. *reniformis*-infected roots

**Figure 4:**
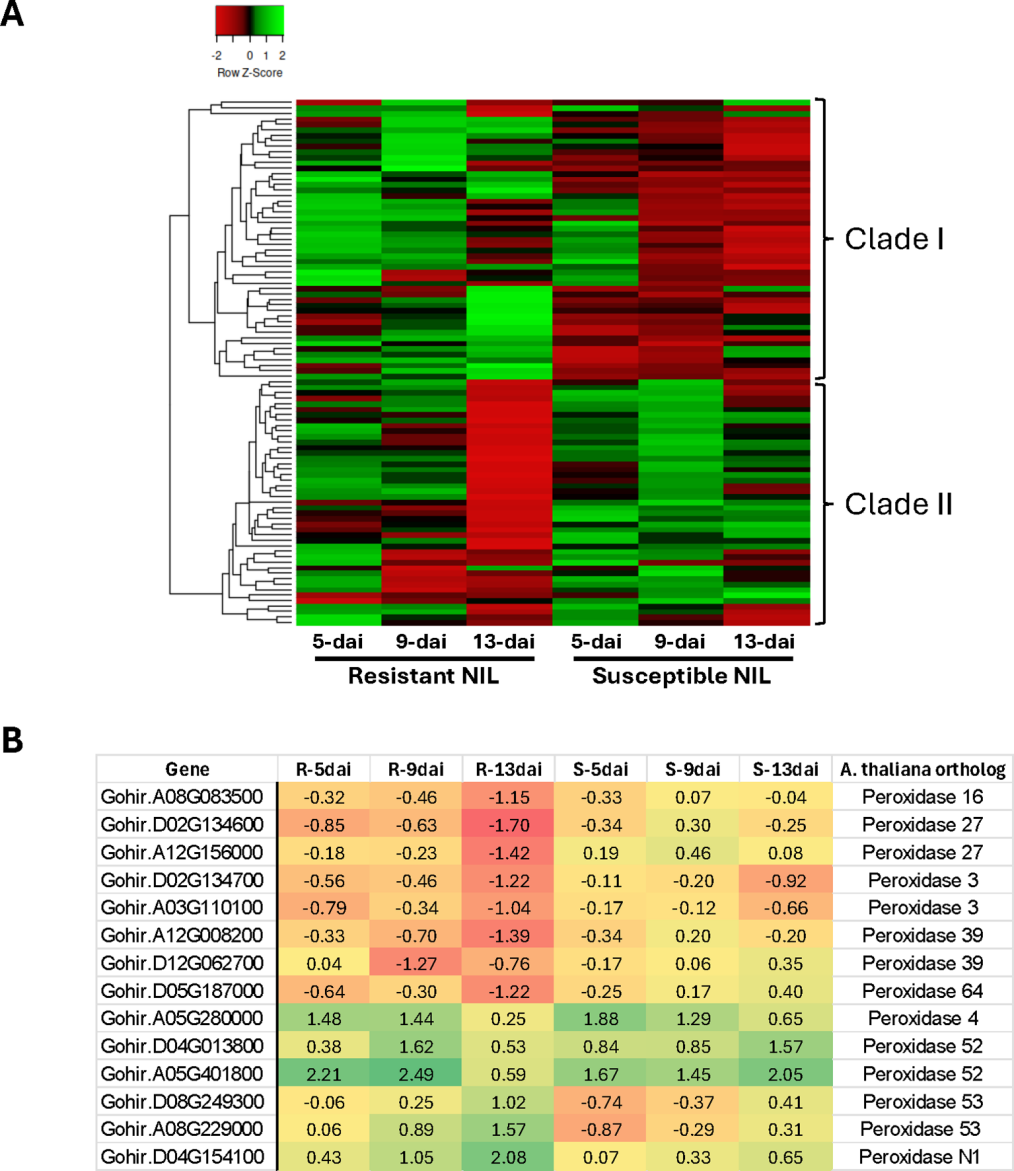
**(A)** Hierarchical cluster analysis of 97 cotton genes having a role in Gene Ontology biological processes oxidation-reduction (GO:0055114) and response to oxidative stress (GO:0006979). **(B)** Log_2_ fold-change expression of peroxidase genes in resistant (R) and susceptible (S) roots at 5-, 9- and 13-dai (days after inoculation) with *Rotylenchulus reniformis*.

Ninety-seven (97) unique genes having a role in GO biological processes oxidation-reduction (GO:0055114) and response to oxidative stress (GO:0006979) were differentially expressed in resistant and susceptible roots following *R*. *reniformis* infection (**Supplementary Table S4**). This group of DEGs was largely represented, in decreasing order of abundance, by peroxidases, 2-oxoglutarate-dependent dioxygenases, laccases, Berberine bridge enzymes, ACC oxidases, and cytochrome P450 enzymes (**Supplementary Table S4**). Two distinct clades/expression profiles emerged when hierarchical clustering of these DEG profiles is presented as a heat map across all timepoints for resistant and susceptible plants (**Figure 4A**). In Clade I, there is a general up-regulation or no change in expression across timepoints in resistant plants but an overall down-regulation in susceptible roots. In contrast, in Clade II, there is a clear trend of significant down-regulation of gene expression at 13-dai in resistant roots versus susceptible where gene expression remains largely unchanged from baseline levels. A closer examination of this 13-dai down-regulated group in resistant roots shows it is partially comprised of multiple peroxidases (**Figure 4B**, **Supplementary Table S4**). A total of 14 peroxidase genes were differentially expressed in resistant roots with more than half showing significant down-regulation at 13-dai. The remaining peroxidase genes show an inverse expression pattern, being up-regulated at various points throughout infection (**Figure 4B**).

### 3.6 Transcription factor analysis

**Figure 5:**
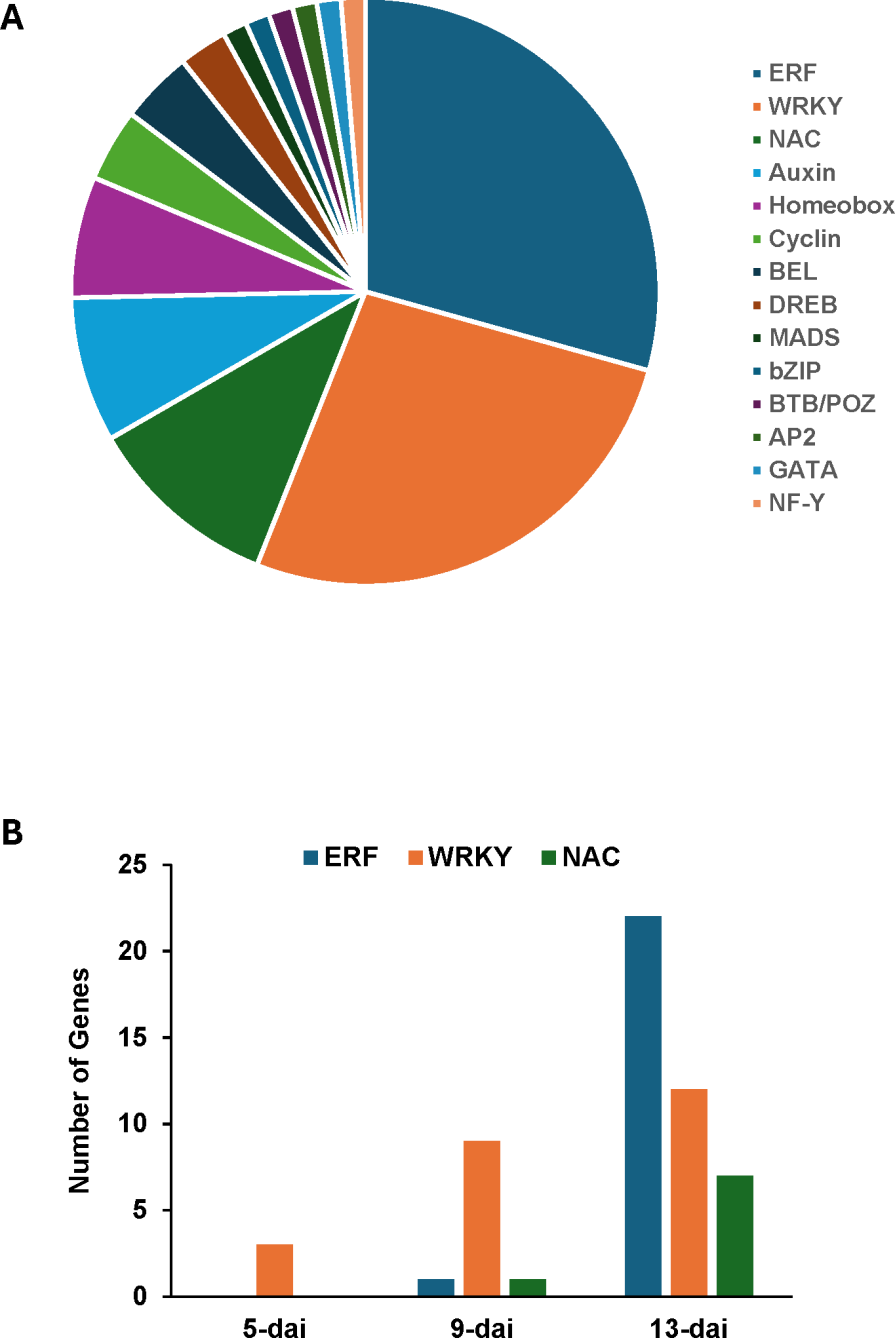
**(A)** Proportional breakdown of 84 cotton genes encoding members of various transcription factor families that were differentially expressed in resistant cotton roots following infection with *Rotylenchulus reniformis*. **(B)** Numbers of ERF (ethylene-responsive factor), WRKY, and NAC transcription factor genes differentially expressed in resistant roots at 5-, 9-, and 13-days after inoculation (dai) with *R*. *reniformis*.

A total of 84 genes representing multiple transcription factor (TF) families were differentially expressed following *R*. *reniformis* infection with the overwhelming majority being in resistant plants (**Supplementary Table S5**). Ethylene-responsive factors and WRKY domain-containing genes comprised more than half of all TFs identified (**Figure 5A**). NAC, auxin-related, and homeobox-related TFs followed in abundance. Numbers of genes corresponding to the ERF, WRKY, and NAC TF families increased steadily over the course of *R*. *reniformis* infection in resistant roots (**Figure 5B**).

Ethylene-responsive transcription factors (ERFs) are involved in multiple signal transduction pathways such as hormone signaling (ethylene, auxin, JA) and different aspects of the cotton defense response (Zafar et al., 2022). Twenty-one (21) genes with significant identity to various ERFs were differentially expressed in resistant roots upon *R*. *reniformis* infection (**Table 3**). Gohir.D03G166600 was the single ERF differentially expressed in susceptible plants being repressed by > 2-fold at 5-dai, while in resistant roots this gene was induced by 4-fold at 13-dai. The remaining 20 ERFs were all induced in resistant plants at 13-dai with the exception of Gohir.A08G189900 which was induced at 9-dai. Cotton genes similar to ERF17 family members were induced at 8-16-fold in resistant plants by 13-dai (**Table 3**). Likewise, Gohir.A13G040200, a homolog of ERF109, was induced by ~16-fold in resistant plants at 13-dai. ERF17 and ERF109 play roles in plant defense signaling including oxidative stress responses and programmed cell death (**Table 3**).

**Table 3:** Cotton ethylene-responsive transcription factor (ERF) differentially expressed genes in response to reniform nematode infection in resistant and susceptible plants.

Gene ID	Treatment^†^	Log_2_(FC)	A.t. ERF homolog	GO: Biological Process^‡^
Gohir.A08G189900	Res-9DAI	1.17	ERF1B	Defense; ET and JA signaling
Gohir.A13G168600	Res-13DAI	1.21	ERF1B	
Gohir.A04G035500	Res-13DAI	1.37	ERF4	Hypoxia; ISR; ET signaling
Gohir.D08G185900	Res-13DAI	2.18	ERF5	ET signaling; auxin response; cold response
Gohir.A08G167100	Res-13DAI	1.67	ERF5	
Gohir.D12G217000	Res-13DAI	1.35	ERF5	
Gohir.A12G214500	Res-13DAI	1.20	ERF5	
Gohir.D03G166600	Res-13DAI	2.00	ERF5	
Gohir.D03G166600	Sus-5DAI	-1.07	ERF5	
Gohir.A09G073300	Res-13DAI	1.16	ERF9	ET signaling; glucosinolates
Gohir.A10G027500	Res-13DAI	1.44	ERF9	
Gohir.A03G155300	Res-13DAI	2.12	ERF9	
Gohir.A06G129800	Res-13DAI	1.94	ERF11	ET signaling; cell division
Gohir.D05G236200	Res-13DAI	1.65	ERF12	ET signaling
Gohir.D06G080300	Res-13DAI	4.22	ERF17	Defense; oxidative stress; JA; wounding
Gohir.D07G016000	Res-13DAI	3.13	ERF17	
Gohir.A06G078800	Res-13DAI	3.25	ERF17	
Gohir.D11G008600	Res-13DAI	2.84	ERF26	ET signaling
Gohir.A11G009300	Res-13DAI	2.22	ERF26	
Gohir.D11G042800	Res-13DAI	1.94	ERF106	ET signaling
Gohir.A13G040200	Res-13DAI	3.93	ERF109	Defense; PCD; auxin response

^†^Resistant (Res) or Susceptible (Sus) cotton genotype.

^‡^ET, ethylene; JA, jasmonic acid; ISR, induced systemic resistance; PCD, programmed cell death.

WRKY transcription factors represented the second major TF class with (17) members being differentially expressed in resistant roots upon *R*. *reniformis* infection (**Table 4**). WRKY genes are transcriptional regulators that are involved in activating and propagating PTI and ETI signaling pathways as well as in response to various abiotic stressors (Javed and Gao, 2023). Similar to the ERF transcription factor class, most of the WRKY genes identified here were induced at a single later timepoint with only four genes being differentially regulated at more than one timepoint. GhWRKY5 was the only gene induced across all timepoints and also the only WRKY gene induced in susceptible plants but only at 5-dai. Three GhWRKY70-like genes were identified with Gohir.D02G003400 being the most up-regulated of all WRKYs discovered at ~ 5-fold. GhWRKY70 has been shown to regulate jasmonic acid production and promote resistance to verticillium wilt (Zhang et al., 2023). Two genes sharing identity with GhWRKY40 were identified that showed induction at 9- and 13-dai. GhWRKY40 has been shown to be regulated by salicylic acid and other hormones while mediating wound- and pathogen-induced responses (Wang et al., 2014). Another regulator of JA signaling, GhWRKY33 (Ji et al., 2023), was identified in our analysis as being induced more than 4-fold at 13-dai.

**Table 4:** Cotton WRKY transcription factor differentially expressed genes in response to reniform nematode infection in resistant and susceptible plants.

Gene ID	Treatment^†^	Log_2_(FC)	TrEMBL Hit (E-value)	WRKY Class^‡^
Gohir.D07G088100	Res-13DAI	1.29	Uncharacterized protein (*G*. *raimondii*) (0.0)	N.d.
Gohir.D03G078600	Res-13DAI	1.37	WRKY transcription factor 12 (*G*. *hirsutum*) (0.0)	IId
Gohir.A06G182900	Res-9DAI	1.19	Probable WRKY transcription factor 40 (*G*. *hirsutum*) (0.0)	I
Gohir.D06G103300	Res-13-DAI	1.04	Probable WRKY transcription factor 40 (*G*. *hirsutum*) (0.0)	I
Gohir.D11G100800	Res-13DAI	1.60	WRKY transcription factor 50 (*G*. *hirsutum*) (0.0)	III
Gohir.D03G046600	Res-5DAI	1.13	Probable WRKY transcription factor 51 (*G*. *hirsutum*) (4.6E-142)	IIb
	Res-9DAI	1.28		
Gohir.A12G235400	Res-13DAI	2.23	WRKY protein 33 (*G*. *hirsutum*) (0.0)	IIc
Gohir.D13G007900	Res-5DAI	1.32	Probable WRKY transcription factor 54 (*G*. *hirsutum*) (0.0)	IId
	Res-9DAI	1.35		
Gohir.D02G003400	Res-9DAI	2.25	Probable WRKY transcription factor 70 (*G*. *hirsutum*) (0.0)	IIa
	Res-13DAI	2.54		
Gohir.A06G180000	Res-9DAI	1.53	Probable WRKY transcription factor 70 (*G*. *hirsutum*) (0.0)	IIa
Gohir.A05G404200	Res-13DAI	1.54	Probable WRKY transcription factor 70 (*G*. *hirsutum*) (0.0)	IIa
Gohir.A05G277500	Res-13DAI	1.22	WRKY protein 27 (*G*. *hirsutum*) (0.0)	III
Gohir.D04G011700	Res-9DAI	1.90	WRKY transcription factor 14 (*G. hirsutum*) (0.0)	I
Gohir.A02G003000	Res-5DAI	1.71	WRKY transcription factor 5 (*G. hirsutum*) (0.0)	III
	Sus-5DAI	1.09		
	Res-9DAI	1.87		
	Res-13DAI	1.63		
Gohir.A10G038400	Res-13DAI	1.22	WRKY transcription factor 92 (*G. hirsutum*) (0.0)	IIc
Gohir.A10G071700	Res-9DAI	1.55	Probable WRKY transcription factor 75 (*G. hirsutum*) (3.4E-120)	N.d.
Gohir.D02G146700	Res-9DAI	1.01	WRKY transcription factor 8 (*G. hirsutum*) (4.2E-94)	IIb

^†^Reniform nematode resistant (Res) or susceptible (Sus) plants.

^‡^ Dou et al. (2014).

### 3.7 Defense response-related DEGs

A number of genes with homology to known defense-related pathways were up-regulated in resistant roots versus susceptible. Cotton genes representative of PR-1, basic and acid endochitinases, thaumatin-like proteins, and MIC-3 were induced across all three timepoints. Multiple genes with similarity to the nematode resistance protein-like HSPRO2 from *Arabidopsis thaliana* were up-regulated in resistant roots and, in some cases, down-regulated in susceptible roots. Gohir.A05G303900 and Gohir.A10G233600 were both induced 2-3-fold in the resistant line at 13-dai but repressed more than 2-fold in the susceptible at the same timepoint. Interestingly, the likely homoeologous copies of these genes, Gohir.D05G302900 and Gohir.D10G245900 were also induced ~ 3-fold in resistant plants but were not differentially regulated in susceptible roots.

### 3.8 qRT-PCR verification of RNA-Seq results

**Figure 6:**
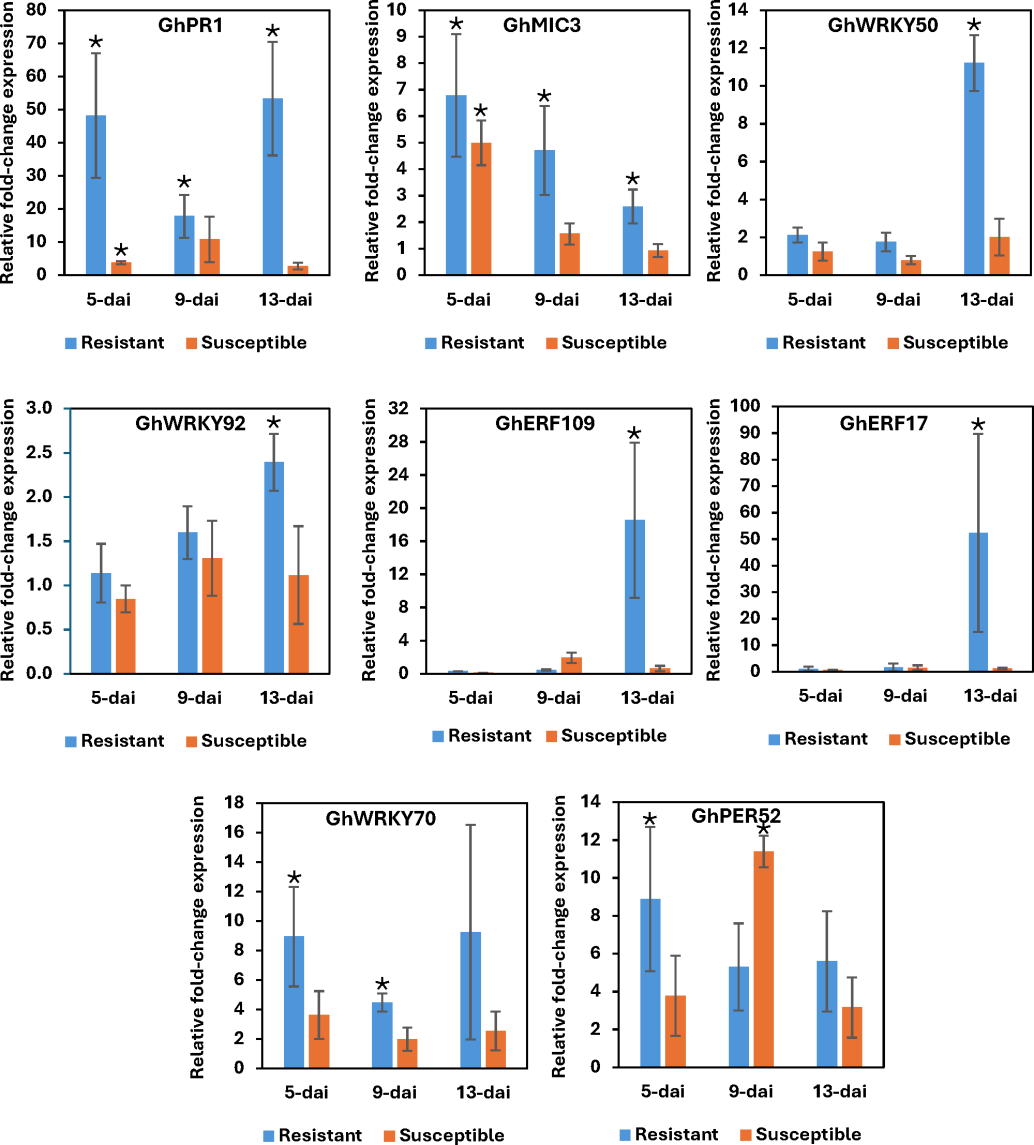
Quantitative RT-PCR of select defense-related genes identified as differentially regulated in resistant and susceptible roots by RNA-Seq following *Rotylenchulus reniformis* infection. Bars represent the mean fold-change (inoculated vs. control) of three biological replicates ± standard error. Asterisks indicate statistically significant fold-change at P ≤ 0.05.

Nine (9) defense-related genes were selected for qRT-PCR to check the validity/accuracy of the RNA-Seq data analyses. The cotton genes GhPR1 and GhMIC3 were both induced across all timepoints in resistant plant roots while both genes were induced only at 5-dai in susceptible roots (**Figure 6**). These results are consistent with the RNA-Seq analysis with the exception that GhPR1 induction was not detected at such a high level as by qRT-PCR. This likely reflects the difference in sensitivity between the assays for this particular transcript. qRT-PCR showed that the WRKY genes GhWRKY50 and GhWRKY92 were both induced at 13-dai in resistant roots but not differentially expressed at all in the susceptible line (**Figure 6**). Likewise, the ERF genes GhERF109 and GhERF17 showed dramatic induction in resistant roots at 13-dai, whereas no significant change in expression was observed in susceptible roots (**Figure 6**). The WRKY gene GhWRKY70 was induced at all timepoints, but significantly so at 5- and 9-dai (**Figure 6**). This expression profile correlates well with that determined by RNA-Seq (**Table 4**). The peroxidase gene GhPER52 was also assessed by qRT-PCR. RNA-Seq showed early induction of this gene in resistant roots and somewhat later induction in the susceptible (**Figure 4B**), and in this assay, induction of GhPER52 was at significant levels in the resistant line at 5-dai (**Figure 6**). GhPER52 was significantly induced in susceptible roots at 9-dai. Overall, the qRT-PCR expression profiles of the selected genes mirrored those provided by RNA-Seq analysis.

### 3.9 RNA-Seq SNP identifies candidate *Ren^barb2^* QTL resistance genes

The *Ren^barb2^* QTL is defined by the SSR markers BNL3279 and BNL4011 on chromosome 21 (D11) (Gutierrez et al., 2011). Using the TX-JGIv1.1 genome sequence, these markers delineated a ~ 1.2 Mbp region, bp 65886069 – 67094220, that contained 59 predicted genes. Using all of the available RNA-Seq data from the resistant root samples, we detected 25 transcripts that contained SNPs compared to the TX-JGIv1.1 genome (**Supplementary Table S6**). Furthermore, we were able to identify non-synonymous SNPs specific to the resistant line that occurred within the coding region of 16/25 genes whose expression we detected. We were also able to determine that the non-synonymous SNPs for 3/16 genes were shared within the genomic sequence of the B713 cotton line that also contains the *Ren^barb2^* QTL (Perkin et al., 2021). The non-synonymous SNPs within the remaining 13/16 genes were found to be present within the *R*. *reniformis*-susceptible *G*. *barbadense* genome sequence 3-79 HAUv2 and were dropped from further study. The gene IDs and SNP position data for the remaining candidate *Ren^barb2^* resistance genes, based on our RNA-Seq SNP analysis of resistant roots, are provided in **Table 5**.

**Table 5:** Candidate reniform nematode resistance genes within the *Ren^barb2^* mapping interval with protein variants absent in susceptible *Gossypium barbadense* protein database yet present in the *Ren^barb2^*-resistant B713 genome.

Gene ID	Description	Position†	Ref	Alt	Protein variant	Affected domain‡
Gohir.D11G301700	Rust resistance kinase Lr10 (*Gossypium hirsutum*)	66047535	G	C	F64L	Pfam13947
Gohir.D11G302300	Putative disease resistance protein At1g5018 (*Gossypium hirsutum*)	66163512	C	T	S556F	N.d.
		66163743	C	G	P479R	N.d.
		66163981	T	C	S400P	Pfam00931
		66164023	A	G	N386D	Pfam00931
		66164083	T	G	L366V	Pfam00931
		66172269	A	G	S209G	Pfam00931
Gohir.D11G306000	Cold-responsive protein kinase 1 (*Gossypium hirsutum*)	67088348	G	A	G284R	Cd14066

^†^Position on chromosome 21(D11) of TX-JGIv1.1 assembly.

^‡^pfam13947 – wall associated receptor kinase galacturonan-binding; pfam00931 – NB-ARC domain; cd14066 – Ser/Thr kinase catalytic domain; N.d. – no domain detected.

Gohir.D11G301700, having similarity to the Lr10 rust resistance kinase, showed a single non-synonymous SNP that results in a F64L change within the predicted galacturonan-binding region (**Table 5**). A single SNP was also identified within Gohir.D11G306000, a cold-responsive protein kinase homolog, that causes a G284R change within the predicted Ser/Thr kinase catalytic domain. The third candidate gene, Gohir.D11G302300, a predicted CC-NBS-LRR resistance protein, contained six non-synonymous SNPs, with four of the SNPs causing mutations within the predicted NB-ARC domain of the protein.

In addition to the genes listed in **Table 5**, gene Gohir.D11G304100, which encodes a probable LRR-RLK, contained a non-synonymous SNP absent in the susceptible 3-79 HAUv2 genome sequence; however, the SNP was also absent from the resistant B713 genome sequence. Gene Gohir.D11G304600, which lacks homology to any known protein, contained two non-synonymous SNPs but extensive BLAST analyses failed to identify a *G*. *barbadense* homolog (data not shown).

**Figure 7:**
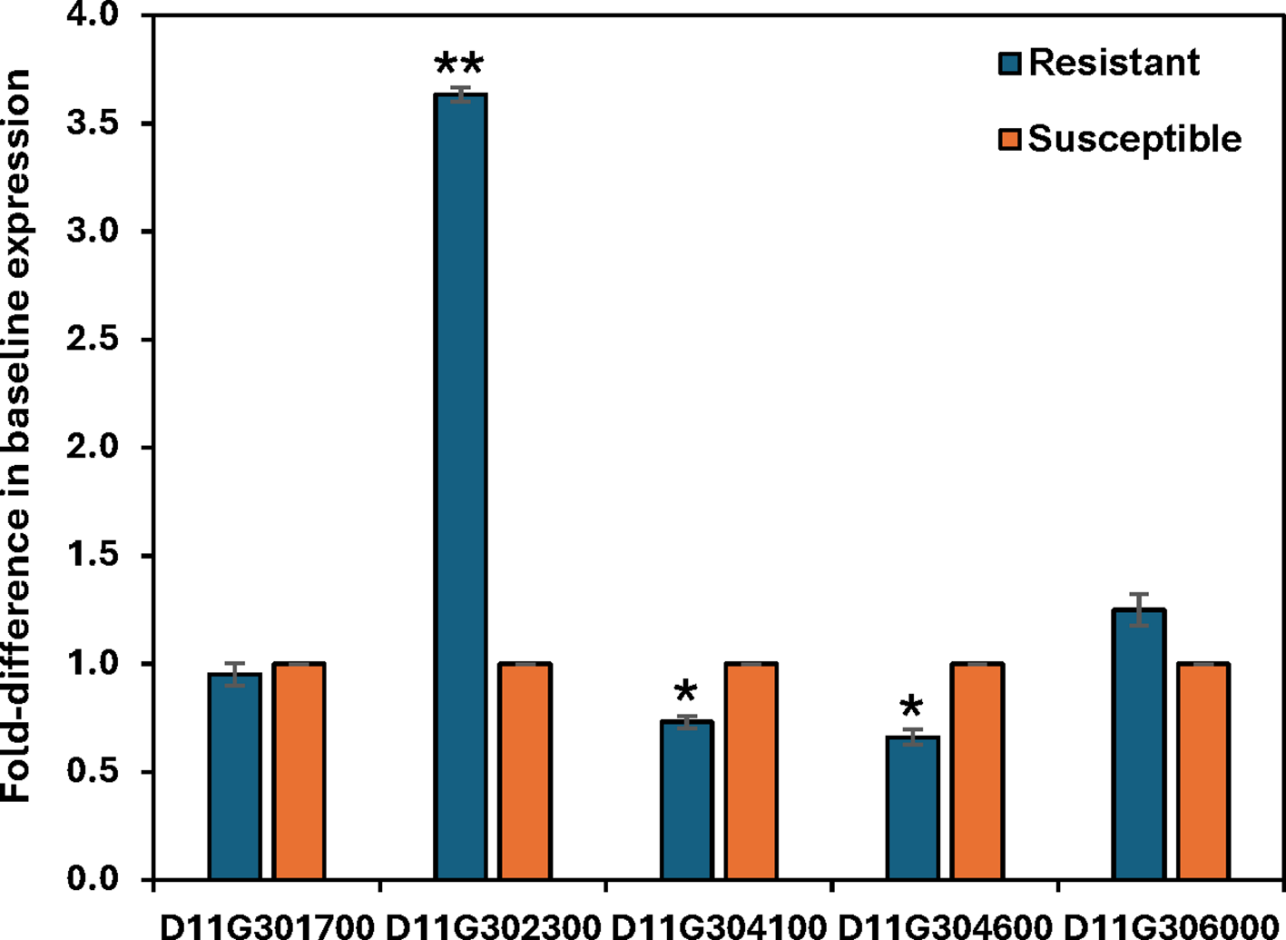
Relative basal expression of five *Ren^barb2^* candidate genes, as measured by quantitative RT-PCR, in control root tissues of resistant plants versus control roots of the susceptible nearly-isogenic line whose value is set equal to 1.0. Bars represent the mean basal fold-difference in resistant vs susceptible roots of three biological replicates ± standard error. Asterisks indicate statistically significant fold-change at *P ≤ 0.05 or **P ≤ 0.001.

We did not detect differential expression of any of the resistance candidate genes in the RNA-Seq data; therefore, qRT-PCR was conducted to detect possible changes in expression. No significant change in transcript levels was observed for any of the candidate genes or for genes D11G304100 and D11G304600 in response to *R*. *reniformis* infection (data not shown). However, significant differences in baseline expression between resistant and susceptible control roots for some of the candidates was observed (**Figure 7**). D11G304100 and D11G304600 both showed slight but significant down-regulation in resistant roots compared to susceptible plants; however, D11G302300, which encodes a putative CC-NBS-LRR resistance protein, showed a nearly 4-fold increase in expression in control resistant roots versus control susceptible roots (**Figure 7**).

## 4 Discussion

Resistance of cotton to the reniform nematode has been identified in multiple diploid and allotetraploid species; however, the successful introgression of resistance into agronomically viable upland genetic backgrounds has been relatively limited. The wild *G*. *barbadense* accession GB713 has been used as the starting material for developing *R*. *reniformis* resistant upland cotton germplasm multiple times (McCarty et al., 2013; Bell et al., 2015; McCarty et al., 2017). Specifically, the *Ren^barb2^* locus on chromosome 21, which mediates ≥ 80% of GB713-derived resistance, has recently become instrumental in providing cotton producers with effective host plant resistance to *R*. *reniformis* (Wubben et al., 2017; Gaudin et al., 2020; Singh et al., 2023). In addition, molecular markers indicate that alleles of *Ren^barb2^* underly *R*. *reniformis* resistance identified in other wild *G*. *barbadense* accessions (Gaudin and Wubben, 2021). While the genetic inheritance of this resistance has been well studied and documented, the molecular signaling events that work to manifest *Ren^barb2^*-mediated resistance remain unclear, as well as the identity of the causal gene(s) at the *Ren^barb2^* locus. In this report, using nearly isogenic lines that differ only in the presence or absence of *Ren^barb2^*, we provide (i) a comprehensive assessment of transcriptome changes over time in infected resistant and susceptible roots and (ii) a SNP analysis of expressed genes within the *Ren^barb2^* mapping interval.

We discovered a total of 1,099 unique DEGs in resistant and susceptible roots over three timepoints of *R*. *reniformis* infection. More than 88% of the DEGs were from resistant roots with the majority of them appearing at the latest timepoint. The trend of increasing numbers of DEGs in resistant roots over time lies in stark contrast to the significantly decreasing number of *R*. *reniformis* females physically present on the same roots within the same timeframe. This would suggest a signaling cascade is taking place after the initial recognition of the nematode and subsequent ‘early’ response that, in this study, included the induction of biological processes such as lignin catabolism and oxidation/reduction reactions. At 13-dai, when we observed only a handful of sedentary females remaining on resistant roots, there had been a concomitant induction of multiple transcription factor families related to defense responses.

Host resistance to plant-parasitic nematodes (PPNs) involves the perception of nematode-derived molecules such as pathogen-associated molecular patterns (PAMPs) and damage-associated molecular patterns (DAMPs), with both leading to pattern-triggered immunity (PTI) (Sato et al., 2019; Goverse and Mitchum, 2022). PPNs also secrete effectors from their esophageal glands whose function is to modulate host cell gene expression and metabolism and also actively suppress the host plant resistance responses associated with PTI and ETI (Goverse and Mitchum, 2022). PTI-induction can be considered as the first line of defense and involves the initial rapid but transient activation of down-stream defense signaling and defense gene expression. In contrast, ETI-associated defense responses are specific to particular pathogens and can amplify and extend defenses activated by PTI leading to complete resistance (Yu et al., 2024). The categories of DEGs identified in *Ren^barb2^*-resistant and susceptible NILs over a time-course of *R*. *reniformis* infection strongly suggest the triggering of ETI-mediated defense signaling in resistant plants but also a short-lived, much weaker, early defense response in susceptible roots that resembles PTI. For example, both resistant and susceptible roots showed induction of oxidation-reduction genes at 5-dai, with more in the resistant, representing an initial defense response involving the production and regulation of reactive oxygen species (ROS). However, these genes are not found in susceptible roots by the next timepoint but they remain up-regulated in resistant roots at 9-dai.

The *MIC-3* gene family was originally identified as a root-specific 14-kDa protein that accumulated specifically within the immature galls of root-knot nematode (RKN; *Meloidogyne incognita*) resistant plants having the qMi-C11 and qMi-C14 QTL at an early timepoint in the RKN infection process (Callahan et al., 1997; Zhang et al., 2002; Wubben et al., 2008). In this study, we discovered that *MIC-3* was induced in *R*. *reniformis* resistant plants across 5-, 9-, and 13-dai but only at 5-dai in the susceptible genotype. This finding lends credence to the hypothesis that the *MIC-3* gene family is a cotton-specific and root-specific collection of defense genes that are coregulated along with other SA-mediated pathways, e.g., PR-1, and are likely activated by classical resistance genes. However, it is unlikely that *MIC-3* plays a direct role in *Ren^barb2^*-mediated resistance to *R*. *reniformis* since cotton lines overexpressing MIC-3, in contrast to RKN, showed no decrease in *R*. *reniformis* reproduction (Wubben et al., 2015).

Three genes were discovered that are homologous to the nematode resistance protein HSPRO2 from *Arabidopsis thaliana*. Previous work demonstrated that HSPRO2, in combination with AtWRKY53, was a positive regulator of basal resistance against *Pseudomonas syringae* (Murray et al., 2007). In our experiment, HSPRO2-like genes were up-regulated in resistant plants at 13-dai and for two of the three genes, A10G233600 and A05G303900, they were actively down-regulated in susceptible plants at the same time-point. Furthermore, GhWRKY33 was significantly up-regulated in resistant plants at 13-dai as well, and this gene is homologous to AtWRKY53. It is tempting to speculate that a comparable relationship between these genes exists in upland cotton as in *A*. *thaliana* in regard to reniform nematode resistance.

Upland cotton chromosome 21 (D11) and its homeolog, chromosome 11 (A11), have been identified numerous times as harboring resistance QTL to plant-parasitic nematodes. The *R*. *reniformis* resistance QTL studied in this report, *Ren^barb2^*, has been localized to chromosome 21 and tightly linked to the SSR marker BNL3279 (Gutierrez et al., 2011; Wubben et al., 2017). High level *R*. *reniformis* resistance introgressed from the diploid *G*. *longicalyx* mediated by the *Ren^lon^* QTL was also found to be closely associated with a BNL3279 allele on chromosome 11 (Dighe et al., 2009). Similarly, *R*. *reniformis* resistance derived from the diploid species *G*. *aridum*, known as *Ren^ari^*, was placed on chromosome 21 and linked to the BNL3279 marker (Romano et al., 2009). *Ren^barb2^*, *Ren^lon^*, and *Ren^ari^* have all been shown to be inherited as putative single genes in a dominant fashion (Dighe et al., 2009; Romano et al., 2009; Gutierrez et al., 2011). The shared characteristics of physical location, mode of inheritance, and early action of the resistance phenotypes make it tempting to speculate that *Ren^barb2^*, *Ren^lon^*, and *Ren^ari^* represent alleles of the same *R*. *reniformis* resistance gene or, at the minimum, represent members of a tightly linked cluster of classical nematode R-genes.

SNP analysis of RNA-Seq data from the present study of *Ren^barb2^* plants identified gene D11G302300 which encodes a predicted CC-NBS-LRR protein within the established QTL interval, and which possesses multiple non-synonymous mutations within the NB-ARC and LRR domains. This gene also showed a significantly higher level of baseline expression in the resistant NIL compared to the susceptible line. Interestingly, in a recent comparative genome analysis of multiple germplasm lines having the *Ren^barb2^* resistance QTL, a pair of NB-ARC domain containing genes were identified within a structural variant on chromosome 21, specific to the *Ren^barb2^* QTL region, that showed constitutive up-regulated gene expression compared to *R*. *reniformis* susceptible lines (Cohen et al., 2024). It is possible that the increased resistant basal expression we discovered for D11G302300 may reflect the combined expression of the NB-ARC paralogs identified by Cohen et al. (2024). Further comparative genomic and functional analyses of D11G302300, and other candidates identified in this study, will not only shed light on the causal *Ren^barb2^* gene but also likely provide valuable information about the genes underlying *Ren^lon^* and *Ren^ari^* resistance mechanisms.

## Data Availability

The datasets presented in this study can be found in online repositories. The names of the repository/repositories and accession number(s) can be found below: https://www.ncbi.nlm.nih.gov/, PRJNA1184828.
