# Supplementary material for: Transcriptome profiling and RNA-Seq SNP analysis of reniform nematode (Rotylenchulus reniformis) resistant cotton (Gossypium hirsutum) identifies activated defense pathways and candidate resistance genes
Source: Front Plant Sci. 2025 Feb 19;16:1532943. doi: 10.3389/fpls.2025.1532943 (PMC11879972; doi:10.3389/fpls.2025.1532943)
Supplement: Supplementary file 2 [file Table2.docx]

| **Table S1.** Candidate and defense gene quantitative RT-PCR primers used in this study. | | |
| --- | --- | --- |
| **Gene** | **Forward primer (5'→3')** | **Reverse primer (5'→3')** |
| D11G301700 | GGCTCGACTACTTACATCAAGG | GCAAGACCGAAATCAGCAAC |
| D11G302300 | CAGAGTTGCCATCATCTCTAGG | GCTCCATCCTCCATATCACATT |
| D11G304100 | CTCAGATTGCAACACAGATGAAG | GCTCACCACCGAAGACATTA |
| D11G304600 | GGATAATGGGCCTTAGTTATTTAGGT | CCCACTCAGAACAAGACCAAC |
| D11G306000 | AATGTGATCGCCGACATAGAG | GAGTTTGTGCAAGGCTGTTATT |
| D10G244600 [SNC1] | GACACGTTAGAGAAGCAGGAA | CCACTTATGTCCTCCAGGTAAC |
| A12G030800 [PR1] | GAAGGCCGACTACGATTACAG | CACAACCAAGATGGACAGAGT |
| A10G038400 [WRKY92] | CAGGTTTCTGACTATGGGCTAC | AAGAAGCGGAAAGAATGGGATA |
| D11G100800 [WRKY50] | TATATCGCCGGCAACATCTG | GGTCTCCGATTGGCGAATTA |
| A04G019400 [MIC3] | CTATCAGTTTGTGCTGTGTGAC | GGTAGAAGAAGTAGGAATCCAGTAG |
| GhUBQ14 | CAACGCTCCATCTTGTCCTT | TGATCGTCTTTCCCGTAAGC |
