# Supplementary material for: Transcriptome profiling and RNA-Seq SNP analysis of reniform nematode (Rotylenchulus reniformis) resistant cotton (Gossypium hirsutum) identifies activated defense pathways and candidate resistance genes
Source: Front Plant Sci. 2025 Feb 19;16:1532943. doi: 10.3389/fpls.2025.1532943 (PMC11879972; doi:10.3389/fpls.2025.1532943)
Supplement: Supplementary file 6 [file Table6.docx]

| **Gene** | **No. snps** | **Syn/Non-syn** | **G.b. proteindb**  **present/not present** |
| --- | --- | --- | --- |
| Gohir.D11G300100 | 5 | 5/0 | NA |
| Gohir.D11G300200 | 3 | 3/0 | NA |
| Gohir.D11G300500 | 4 | 2/2 | 2/0 |
| Gohir.D11G301000 | 2 | 2/0 | NA |
| Gohir.D11G301300 | 8 | 2/6 | 6/0 |
| Gohir.D11G301400 | 1 | 1/0 | NA |
| Gohir.D11G301500 | 8 | 6/2 | 2/0 |
| Gohir.D11G301700 | 3 | 0/3 | 2/1 |
| Gohir.D11G301900 | 3 | 3/0 | NA |
| Gohir.D11G302100 | 3 | 2/1 | 1/0 |
| Gohir.D11G302300 | 8 | 2/6 | 0/6 |
| Gohir.D11G302500 | 5 | 4/1 | 1/0 |
| Gohir.D11G302700 | 6 | 4/2 | 2/0 |
| Gohir.D11G302800 | 2 | 1/1 | 1/0 |
| Gohir.D11G303100 | 1 | 0/1 | 1/0 |
| Gohir.D11G303500 | 1 | 1/0 | NA |
| Gohir.D11G304100 | 1 | 0/1 | 0/1 |
| Gohir.D11G304200 | 5 | 5/0 | NA |
| Gohir.D11G304600 | 2 | 0/2 | no Gb homologue |
| Gohir.D11G305200 | 2 | 0/2 | 2/0 |
| Gohir.D11G305300 | 1 | 0/1 | 1/0 |
| Gohir.D11G305400 | 1 | 1/0 | NA |
| Gohir.D11G305500 | 2 | 2/0 | NA |
| Gohir.D11G305900 | 4 | 1/3 | 3/0 |
| Gohir.D11G306000 | 3 | 2/1 | 0/1 |
