# Supplementary material for: Transcriptome profiling and RNA-Seq SNP analysis of reniform nematode (Rotylenchulus reniformis) resistant cotton (Gossypium hirsutum) identifies activated defense pathways and candidate resistance genes
Source: Front Plant Sci. 2025 Feb 19;16:1532943. doi: 10.3389/fpls.2025.1532943 (PMC11879972; doi:10.3389/fpls.2025.1532943)
Supplement: Supplementary file 7 [file Presentation1.pptx]

## Slide 1
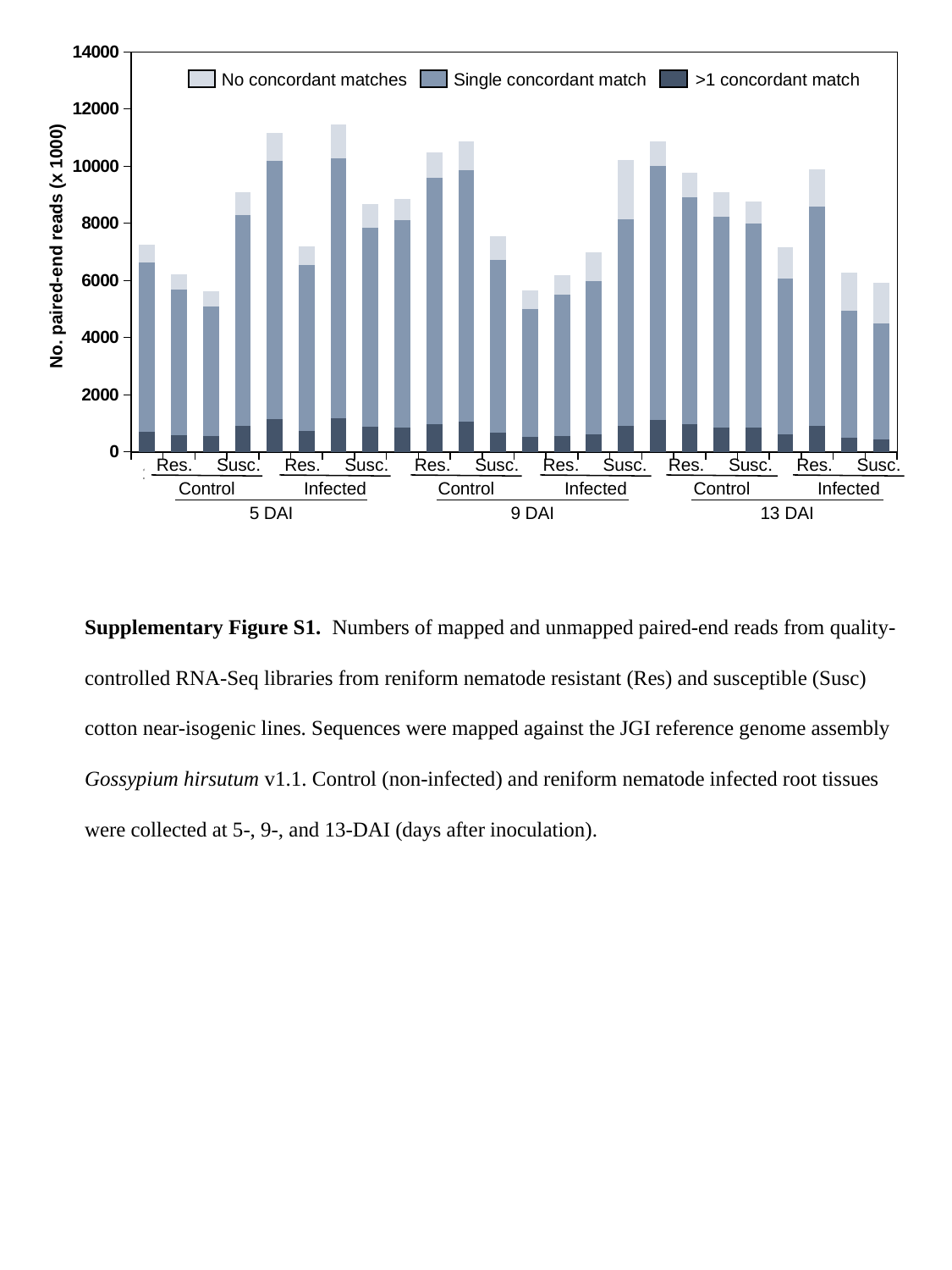

### Chart
| Category | Multiple concordant matches | Single concordant match | No concordant matches |
|---|---|---|---|
| 1 | 696.723 | 5927.222 | 640.344 |
| 2 | 593.973 | 5091.32 | 535.498 |
| 3 | 550.668 | 4546.473 | 514.591 |
| 4 | 898.766 | 7379.452 | 797.89 |
| 5 | 1146.03 | 9043.34 | 980.56 |
| 6 | 741.098 | 5814.975 | 632.695 |
| 7 | 1168.236 | 9112.136 | 1190.724 |
| 8 | 885.673 | 6951.526 | 853.884 |
| 9 | 839.535 | 7280.082 | 747.603 |
| 10 | 980.812 | 8600.923 | 910.166 |
| 11 | 1050.035 | 8813.563 | 1005.541 |
| 12 | 688.786 | 6044.089 | 810.865 |
| 13 | 518.259 | 4475.411 | 652.942 |
| 14 | 558.551 | 4935.467 | 696.637 |
| 15 | 627.645 | 5356.277 | 1002.677 |
| 16 | 903.621 | 7232.967 | 2096.325 |
| 17 | 1113.205 | 8902.578 | 864.259 |
| 18 | 982.371 | 7934.048 | 859.193 |
| 19 | 858.823 | 7374.152 | 850.556 |
| 20 | 843.789 | 7138.481 | 773.402 |
| 21 | 616.83 | 5450.334 | 1085.026 |
| 22 | 904.862 | 7668.381 | 1328.549 |
| 23 | 499.338 | 4442.766 | 1323.382 |
| 24 | 446.123 | 4050.32 | 1427.122 |
No concordant matches
Single concordant match
>1 concordant match
No. paired-end reads (x 1000)
Res.
Susc.
Res.
Susc.
Res.
Susc.
Res.
Susc.
Res.
Susc.
Res.
Susc.
Control
Control
Infected
Infected
Control
Infected
5 DAI
9 DAI
13 DAI
Supplementary Figure S1. Numbers of mapped and unmapped paired-end reads from quality-controlled RNA-Seq libraries from reniform nematode resistant (Res) and susceptible (Susc) cotton near-isogenic lines. Sequences were mapped against the JGI reference genome assembly Gossypium hirsutum v1.1. Control (non-infected) and reniform nematode infected root tissues were collected at 5-, 9-, and 13-DAI (days after inoculation).
