# Supplementary material for: Transcriptome profiling and RNA-Seq SNP analysis of reniform nematode (Rotylenchulus reniformis) resistant cotton (Gossypium hirsutum) identifies activated defense pathways and candidate resistance genes
Source: Front Plant Sci. 2025 Feb 19;16:1532943. doi: 10.3389/fpls.2025.1532943 (PMC11879972; doi:10.3389/fpls.2025.1532943)
Supplement: Supplementary file 8 [file Presentation2.pptx]

## Slide 1
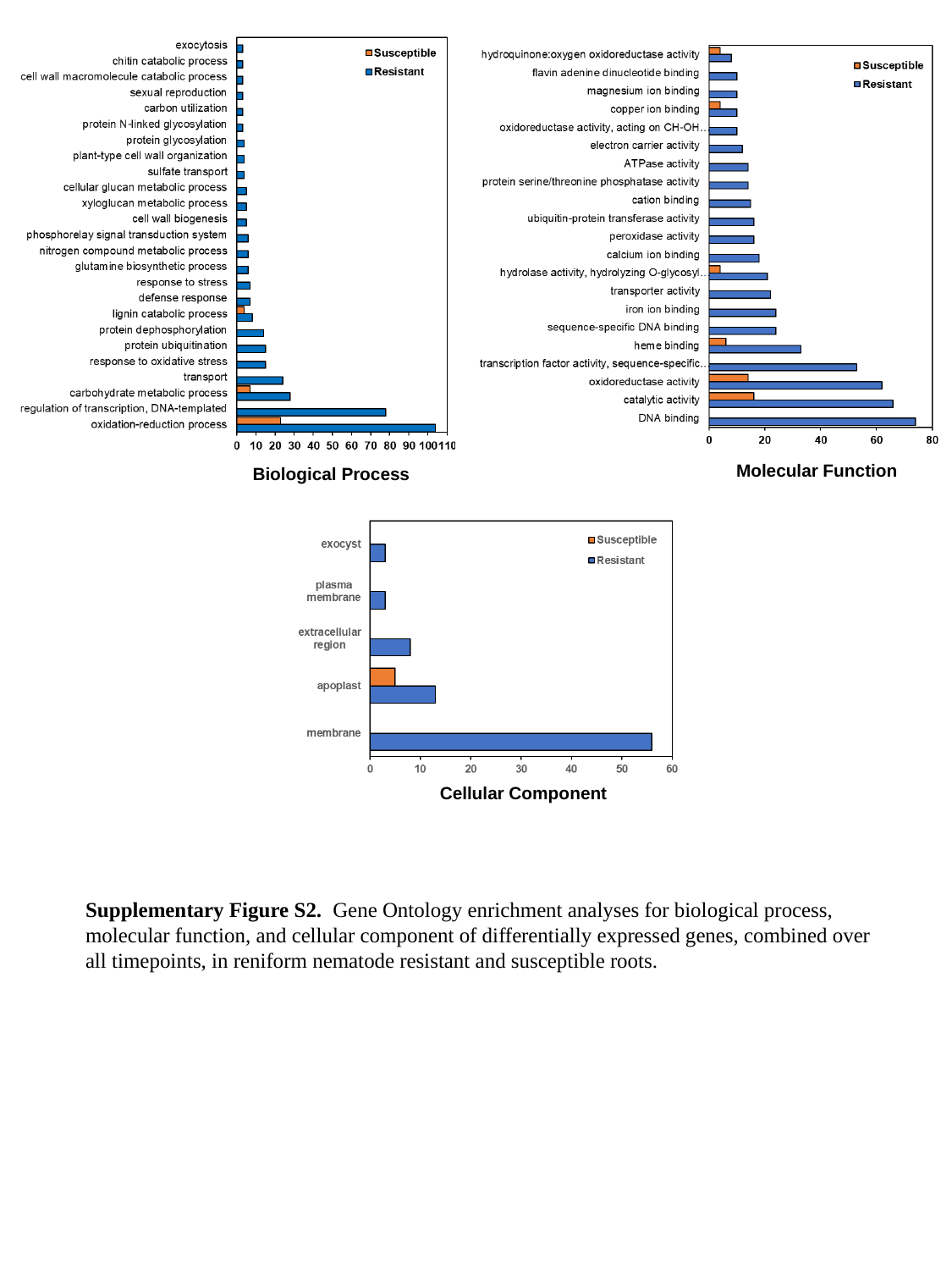

Molecular Function
Biological Process
Cellular Component
Supplementary Figure S2. Gene Ontology enrichment analyses for biological process, molecular function, and cellular component of differentially expressed genes, combined over all timepoints, in reniform nematode resistant and susceptible roots.
